# Prevalence of *Borrelia burgdorferi *sensu lato and *Anaplasma phagocytophilum *in questing *Ixodes ricinus *ticks in relation to the density of wild cervids

**DOI:** 10.1186/1751-0147-51-47

**Published:** 2009-11-27

**Authors:** Olav Rosef, Algimantas Paulauskas, Jana Radzijevskaja

**Affiliations:** 1 Telemark University College, Bø i Telemark, Norway; 2 Vytautas Magnus University, Kaunas, Lithuania

## Abstract

**Background:**

*Borrelia burgdorferi *sensu lato and *Anaplasma phagocytophilum *have been considered as pathogens in animals and humans. The role of wild cervids in the epidemiology is not clear. We analyzed questing *Ixodes ricinus *ticks collected in spring for these pathogens from sites with high (Fjelløyvær and Strøm) and low density (Tjore, Hinnebu and Jomfruland) of wild cervids to study the spread of the pathogens in questing ticks.

**Methods:**

For detection of *Anaplasma phagocytophilum *a 77-bp fragment in the *msp*2 gene was used. Detection of *Borrelia burgdorferi *sensu lato was performed using the FL6 and FL7 primers according to sequences of conserved regions of the *fla *gene. The *Osp*A gene located on the linear 49-kb plasmid was used as target in multiplex PCR for genotyping. Genospecies-specific primers were used in the PCR for *Borrelia burgdorferi *sensu stricto, *B. afzelii *and *B. garinii*.

**Results:**

Infection rates with *Borrelia *spp. were significantly lower at Fjelløyvær and Strøm compared to Tjore and Hinnebu; Fjelløyvær vs. Tjore (χ^2 ^= 20.27, p < 0.0001); Fjelløyvær vs. Hinnebu (χ^2 ^= 24.04, p < 0.0001); Strøm vs. Tjore (χ^2 ^= 11.47, p = 0.0007) and Strøm vs. Hinnebu (χ^2 ^= 16.63, p < 0.0001). The *Borrelia *genospecies were dominated by. *B. afzelii *(82%) followed by *B. garinii *(9.7%) and *B. burgdorferi *sensu stricto (6.9%). *B. burgdorferi *s.s. was only found on the island of Jomfruland. The infection rate of *Anaplasma phagocytophilum *showed the following figures; Fjelløyvær vs Hinnebu (χ^2 ^= 16.27, p = 0.0001); Strøm vs. Tjore (χ^2 ^= 13.16, p = 0.0003); Strøm vs. Hinnebu (χ^2 ^= 34.71, p < 0.0001); Fjelløyvær vs. Tjore (χ^2 ^= 3.19, p = 0.0742) and Fjelløyvær vs. Støm (χ^2 ^= 5.06, p = 0.0245). Wild cervids may serve as a reservoir for *A. phagocytophilum*. Jomfruland, with no wild cervids but high levels of migrating birds and rodents, harboured both B. *burgdorferi *s.l. and *A. phagocytophilum *in questing *I. ricinus *ticks. Birds and rodents may play an important role in maintaining the pathogens on Jomfruland.

**Conclusion:**

The high abundance of roe deer and red deer on the Norwegian islands of Fjelløyvær and Strøm may reduce the infection rate of *Borrelia burgdorferi *sensu lato in host seeking *Ixodes ricinus*, in contrast to mainland sites at Hinnebu and Tjore with moderate abundance of wild cervids. The infection rate of *Anaplasma phagocytophilum *showed the opposite result with a high prevalence in questing ticks in localities with a high density of wild cervids compared to localities with lower density.

## Background

Lyme disease, an important arthropod-borne disease of humans in the northern hemisphere, can manifest in many organ systems with symptoms including skin rashes, meningitis, optic neuritis, facial nerve palsy and atrioventricular nodal block. Failure to treat infection promptly and adequately can result in long-term debilitating effect on the patient's health. Three species have been proven to be pathogenic in humans: *Borrelia afzelii, B. garinii *and *B. burgdorferi *sensu stricto [1]. These species appear to be responsible for causing different clinical syndromes [2].

It is well known that *Ixodes *ticks feed on deer species [3], and that high abundance of *Ixodes *ticks follows a high abundance of deer [4], but the role of cervid species in the epidemiology of Lyme disease is not completely understood. Although it has been suggested that adaptive immune responses may be involved in the regulation of spirochete transmission [5], the detailed mechanisms underlying differential transmission of the *Borrelia *genospecies by hosts are unknown. Investigators have concluded that roe deer (*Capreolus capreolus*) [6] and red deer (*Cervus elaphus*) [7-9] are incompetent reservoirs for *B. burgdorferi*. Spirochaetes that are sensitive to destruction by the complement system of a particular host species are lysed early in the midgut of the feeding tick and are thereby eliminated by the host [10]. These findings have led to the hypothesis that the host range of spirochaete strain is restricted by its repertoire of genes that encode ligands with the high binding affinities for complement inhibition [7].

Tick-borne fever caused by *A. phagocytophilum *has been considered a common disease in domestic ruminants along the coast of southern Norway [11]. Several other mammalian species including wild cervids have also been found infected with *A. phagocytophilum *[12]. Stuen et al. [13] found seroprevalences of granulocytic *Ehrlichia *spp. in moose (*Alces alces*) of 43%, red deer 55%, and roe deer 96% from *I. ricinus *infested counties in Norway. A study in Switzerland found serological evidence of granulocytic ehrlichial infection in roe deer [14].

Human granulocytic anaplasmosis (HGA) caused by *Anaplasma phagocytophilum *was first identified in 1990 in a patient who died [15]. HGA is increasingly recognized as an important and frequent cause of fever after tick bite world wide [16], including Scandinavia [17] where *Ixodes *ticks bite humans. Several *Ixodes *spp. including *I. ricinus*, *I. arboricola, I. calidonicus, I. frontais, I. hexagonus, I. lividus, I. persulcatus, I. trianguliceps, I. urinae *and *I. unicavatus *have been found in Fennoscandia [18-20] Most human cases occur between June and August and usually appear as an undifferentiated febrile illness. The incubation period following tick-bite is 7-10 days and symptoms include high fever, rigors, generalized myalgias, severe headacke and malaise [16]. Bjöersdorff et al. [17] found a seroprevalence of 15-20% among 1000 tick-exposed patients (mainly from Sweden and Norway) and concluded a widespread exposure to granulocytic *Ehrlichia *(now *Anaplasma *spp.). In Slovenia 3.2% of *I. ricinus *were infected with *Anaplasma*, and they were 99.8% identical to those previously determined from human patients [21]. The main vector in Europe is *I. ricinus*. In other continents Zhang et al. [22] found a high seroprevalence rate (8.8%) for *A. phagocytophilum *among 365 farm-workers in China and suggested that human infections with these zoonotic bacteria are frequent and largely unrecognized. A seroprevalence between 2.3% and 5.6% was found in different locations in Mongolia and Walder et al. [23] concluded that *A. phagocytophilum *is endemic. Brown et al. [24] confirmed that woodland rodents can maintain *A. phagocytophilum *in Great Britain in the absence of other reservoir hosts which suggests that *I. trianguliceps *is a competent vector.

The aim of the present study was to compare the prevalence of *B. burgdorferi *s.l. and *A. phagocytophilum *in *I. ricinus *ticks in sites with both high and low abundance of roe deer, red deer and moose to evaluate the role of wild cervids in the epidemiology.

## Materials and methods

### Locations and habitats

Tick samples were collected on two islands on the coast of western Norway: at Strøm (N7048360E498426), on the island of Hitra, and on the island of Fjelløyvær (N7059209E504490) close to the main island Hitra and connected by a bridge. Both islands are largely covered with heath and a mixture of deciduous and pine forest. There are no foxes, but sea gulls and raptorial birds are common, and roe deer and red deer densities are high. There are farms on both islands with grass production and grazing cattle and sheep. Tick samples were also collected at three sites along the southern coast of Norway. These included Tjore, a coastal mainland site (N6463382E473032) located in a mixture of farmland and mixed deciduous, pine and spruce forest, and within 100 m outside of a red deer enclosure; Hinnebu (N6493848E469418) situated 30 km from the coast with similar mixed forest but no agriculture or grazing domestic animals; and Jomfruland, an island with agriculture and mixed forest (N6524446E533677). Jomfruland is frequented by many migrating birds and is grazed by sheep and cattle, but contains no wild cervids. Coordinates are given in UTM32 (Euref 89) values.

### Abundance of roe deer, red deer and moose

We used the official municipal hunting statistics for 2007 for each township involved to estimate the numbers of game animals at each site (Table 1). We have defined low density as less than one animal killed per km^2 ^and high density as more than 3.

**Table 1:** Number of animals killed by hunting per km^2 ^(hunting statistics for 2007)

	Red deer	Roe deer	Moose	Total
Fjelløyvær	0.05 (1)^a^	8.62 (181)	0*	8.67
Strøm	1.94 (846)	1.18 (513)	0*	3.12
Hinnebu	0.05 (30)	0.33 (198)	0.31 (194)	0.69
Tjore	0.02 (6)	0.56 (528)	0.17 (194)	0.75
Jomfruland	0**	0**	0*	0

^a^The numbers in parentheses represent the total number of killed animals.

*Moose is absent.

**Red deer and roe deer are absent.

### Tick collection

Questing *I. ricinus *ticks were collected during spring (April-May) 2006-2008 at all five locations using the standard flagging method [25] by drawing a 1 m^2 ^piece of cotton cloth over the vegetation. Ticks attached to the towel were picked with tweezers and placed into 1.5 ml test tube filled with 70% ethanol.

### DNA extraction and detection of Ixodes ricinus

A modified procedure for extracting DNA with ammonium hydroxide solution (2.5%) was performed [26,27]. The lysates were stored at -20°C until use. For *I. ricinus *identification, the lysates were analysed with species-specific primers IxriF and IxriR resulting in a 150 bp segment of the 5.8 srRNA gene [28,29]. This PCR reaction was further used as positive control. DNA bands were stained with ethidium bromide and visualised by UV transillumination (EASY Win32, Herolab, Germany).

### Detection of Borrelia burgdorferi sensu lato

The occurrence of *Borrelia burgdorferi *s.l. in ticks was determined by polymerase chain reaction by using the oligonucleotide primers FL6 and FL7 according to sequences of conserved regions of the *fla *gene [26]. PCR products were resolved by 1.5% agarose gel electrophoresis with addition of ethidium bromide and visualized under UV light (EASY Win32, Herolab, Germany). The achieved specific amplification products of 276 base pairs (bp) were considered a positive result. Negative and positive controls were included in all runs.

### Genotyping of Borrelia burgdorferi sensu lato

The *OspA *gene located on the linear 49-kb plasmid was used as target in multiplex PCR according to Demaershalck et al. [30]. Genospecies-specific primers were used in the PCR for *B. burgdorferi *sensu stricto, *B. afzelii *and *B. garinii*. PCR amplification products were resolved onto 2.0% agarose gel electrophoresis and visualized under UV light. The specific products of 544 bp (*B*. *burgdorferi *s.s.) 345 bp (*B. garinii) *and 189 bp (*B. afzelii*) were considered to represent positive results. Negative and positive controls were included in all runs.

### Detection of Anaplasma phagocytophilum

*I. ricinus *questing ticks were examined for the prevalence of *A. phagocytophilum *by using the species-specific primers ApMSP2f, ApMSP2r, and TaqMan probe ApMsp2p-FAM, as described by Courtney et al. [31]. A 77-bp fragment in the *msp*2 gene of *A. phagocytophilum *was amplified. PCR was performed using TaqMan Master Mix (Applied Biosystems, CA) in a quantitative thermal cycler (iCycler, Bio-Rad Laboratories, Inc., Hercules, CA). Negative and positive controls were included in all runs.

### Statistics

The data were analysed statistically by means of Pearson's χ^2 ^test by using the statistical package STATISTICA for WINDOWS 5.5. We compared the mean isolation rate of *B. burgdorferi *s.l. and *A. phagocytophilum *for 2006-2008 in sites with different densities of wild cervids.

## Results

The highest density of wild cervids was Fjelløyvær followed by Strøm (Table 1). No *Borrelia *was detected in questing ticks in Fjelløyvær, and low values in Strøm during the three year period (Table 2). The infection rates were significantly lower in areas with high density of wild cervids compared to sites with low density: Fjelløyvær vs. Tjore (χ^2 ^= 20.27, p < 0.0001); Fjelløyvær vs. Hinnebu (χ^2 ^= 24.04, p < 0.0001); Strøm vs. Tjore (χ^2 ^= 11.47, p = 0.0007) and Strøm vs. Hinnebu (χ^2 ^= 16.63, p < 0.0001). There were significantly lower values on Fjelløyvær vs. Jomfruland (χ^2 ^= 10.66, p = 0.0011); Fjelløyvær vs Strøm (χ^2 ^= 4.26, p = 0.0390) and Hinnebu vs. Jomfruland (χ^2 ^= 6.56, p = 0.0104), but no significant difference between Tjore vs. Jomfruland (χ^2 ^= 3.2, p = 0.0735); Strøm vs. Jomfruland (χ^2 ^= 3.24, p = 0.0719) and Hinnebu vs. Tjore (χ^2 ^= 0.27, p = 0.6006). The distribution of genospecies is shown in Table 3. *B. afzelii *dominated with 82% followed by *B. garinii *(9.7%) and *B. burgdorferi *s.s. (6.9%). *B. burgdorfereri s.s*. was only found on the island of Jomfruland.

**Table 2:** Prevalence of *Borrelia burgdorferi *sensu lato in questing *Ixodesricinus *ticks 2006, 2007 and 2008.

Locations	Years	Female			Male			Nymph			Total		
		
		N	Prevalence		N	Prevalence		N	Prevalence		N	Prevalence	
		
			n	%		n	%		n	%		n	%
**Hitra, Fjelløyvær**	2008	9	0	0	8	0	0	43	0	0	60	0	0
	2007	23	0	0	27	0	0	30	0	0	80	0	0
	2006	2	0	0	6	0	0	56	0	0	64	0	0
	
											204	0	0

**Hitra, Strøm**	2008	5	0	0	7	1	14.3	40	0	0	52	1	1.9
	2007	40	0	0	32	0	0	16	0	0	88	0	0
	2006	5	1	20	3	0	0	89	3	3.4	97	4	4.1
	
											237	5	2.1

**Tjore**	2008	24	1	4.2	14	0	0	20	2	10	58	3	5.2
	2007	19	2	10.5	23	3	13	38	1	2.6	80	6	7.5
	2006	4	1	25	4	0	0	16	7	43,8	24	8	33.3
	
											162	17	10.5

**Hinnebu**	2008	31	3	9.7	41	3	7.3	7	0	0	79	6	7.6
	2007	52	15	28.8	57	4	7	6	0	0	115	19	16.5
	2006	42	4	9.5	32	4	12.5	32	4	12.5	106	12	11.3
	
											300	37	11.8

**Jomfruland**	2008	29	3	10.3	30	0	0	20	0	0	79	3	3.8
	2007	30	3	10	20	2	10	22	0	0	72	5	6.9
	2006	8	0	0	8	0	0	76	7	9.2	92	5	5.4
	
											243	13	5.3

N = number of tested ticks; n = number of infected ticks

**Table 3:** Borrelia burgdorferi sensu lato genospecies in questing Ixodes ricinus ticks.

Locations	*B.afzelii* n/N (%)	*B.garinii* n/N (%)	***B.burgdorferi *s.s**. n/N (%)	*B.burgdorferi *s.s.+ *B.afzelii* n/N (%)
**Hitra, Strøm**	4/5 (80)	1/5 (20)	0/5 (0)	0/5 (0)
**Tjore**	13/17 (76.5)	4/17 (23.5)	0/17 (0)	0/17 (0)
**Hinnebu**	35/37 (94.6)	2/37 (5.6)	0/37 (0)	0/37 (0)
**Jomfruland**	7/13 (53.8)	0/13 (0)	5/13 (38.5)	1/13 (7.7)

**Total**	59/72 (82)	7/72(9.7)	5/72(6.9)	1/72(1.4)

N = number of tested ticks; n = number of infected ticks; (%) - prevalence of infection

The prevalence of *A. phagocytophilum *infections in questing ticks (Table 4) was significantly higher in localities with high density of wild cervids compared to localities with lower density (Table 1): Fjelløyvær vs. Hinnebu (χ^2 ^= 16.27, p = 0.0001); Fjelløyvær vs. Støm (χ^2 ^= 5.06, p = 0.0245); Strøm vs. Tjore (χ^2 ^= 13.16, p = 0.0003) and Strøm vs. Hinnebu (χ^2 ^= 34.71, p = 0.0000). The figures for Hinnebu vs. Tjore was (χ^2 ^= 5.07, p = 0.0243); Hinnebu vs. Jomfruland (χ^2 ^= 30.73, p = 0.000) and Jomfruland vs. Tjore (χ^2 ^= 10.97, p = 0.0009). There was one exception, with no significant difference between Fjelløyvær and Tjore where a high level of *A. phagocytophilum *was detected in 2008 (χ^2 ^= 3.19, p = 0.0742) (Table 4). There were no significant difference between Strøm and Jomfruland (χ^2 ^= 0.38, p = 0.54), or Fjelløyvær and Jomfruland (χ^2 ^= 3.78, p = 0.0519).

**Table 4:** Prevalence of *Anaplasma phagocytophilum *in questing *Ixodesricinus *ticks 2006, 2007 and 2008.

Locations	Years	Female			Male			Nymphs			Total		
		
		N	Prevalence		N	Prevalence		N	Prevalence		N	Prevalence	
		
			n	%		n	%		n	%		n	%
**Hitra, Fjelløyvær**	2008	9	0	0	8	0	0	42	2	4.8	59	2	3.4
	2007	23	4	17.4	24	3	12.5	30	2	16.7	77	12	15.6
	2006	2	1	50	6	0	0	56	2	3.6	64	3	4.7
	
											200	17	8.5

**Hitra, Strøm**	2008	5	0	0	7	2	28.6	40	11	27.5	52	13	25
	2007	40	9	22.5	35	9	25.7	33	3	9.1	108	21	19.4
	2006	5	1	20	3	1	33.3	89	8	8.9	97	10	10.3
	
											257	44	17.1

**Tjore**	2008	24	3	12.5	14	0	0	20	2	0.8	58	5	8.6
	2007	19	0	0	22	0	0	21	0	0	63	0	0
	2006	4	0	0	4	0	0	16	0	0	24	0	0
	
											145	5	3.4

**Hinnebu**	2008	31	1	3.2	41	0	0	7	0	0	79	1	1.3
	2007	27	0	0	18	0	0	5	0	0	50	0	0
	2006	42	0	0	32	0	0	32	0	0	106	0	0
	
											235	1	0.4

**Jomfruland**	2008	29	6	20.7	40	10	25	57	13	22.4	126	29	23.0
	2007	50	8	16	32	3	9.4	49	4	8.2	131	15	11.5
	2006	8	1	12.5	8	1	12.5	75	6	8	91	8	8.7
	
											348	52	14.9

N = number of tested ticks; n = number of infected ticks

## Discussion

Kurtenbach et al. [5] showed that sera from red deer were indiscriminating borrealicidal for the three human pathogenic strains. The reservoir incompetence of roe deer [32] and red deer [9] correlates with this borrealicidic effect. Complement appears relevant to host incompetency for *Borrelia*, and this carries over to prevent tick infection and lyse the spirochetes early in the midgut of the feeding tick, and are thereby eliminated by the host [10]. Low levels of *B. burgdorferi *s.l. in ticks werre found in both sites on Hitra (Table 2). No infected ticks were detected in Fjelløyvær during the three year period, and only a low level *of B. burgdorferi *s.l. in 2006 (4.1%) and in 2008 (1.9%) at Strøm (Table 2). Fjelløyvær has a very high abundance of roe deer, but red deer are nearly absent (Table 1). Strøm has a high abundance of both red and roe deer. We believe that the main route for the tick cycles is red deer and roe deer at Strøm and Fjelløyvær. The high abundance of deer gives high levels of ticks, but the serum incompetence will reduce both the infection in ticks and the risk of Lyme disease transmission.

This contrasts with the figures at Hinnebu where the infection rates with *B. burgdorferi *s.l. were 10%, 16.5% and 7.6% in 2006-2008 (Table 2). Hinnebu is forest-covered with a low density of moose and roe deer, and a low abundance of red deer. Harvest statistics show a much lower combined density of wild cervids at Hinnebu than at Fjelløyvær and Strøm (Table 1). Tjore has low densities of red deer and moose, and a moderate density of roe deer. Ticks collected outside a fenced red deer farm indicated that the presence of the farm had no influence on the level of *B. burgdorferi *s.l. The overall infection rates in ticks were 33.3% in 2006, 6.9% in 2007 and 5.2% in 2008. The capacity of deer to act as reservoirs for *B. burgdorferi *s.l., is controversial [33,34]. However, our results clearly support the idea that wild cervids are incompetent reservoirs. Our results showed that the infection rates in questing ticks were significantly lower in areas with a high density of wild cervids (Fjelløyvær and Strøm) compared to sites with low density (Tjore and Hinnebu) (Tables 1 and 2).

*B. afzelii *genospecies from ticks dominated with 82% as shown in Table 3. This genospecies is related to rodents [7,35,36]. *B. garinii *was detected in Strøm, Tjore and Hinnebu while *B. burgdorferi *s.s was found on questing ticks from Jomfruland. Though Jomfruland has no wild cervids, it does have grazing domestic animals, plus migrating birds during spring and autumn. In this site we investigated 49 *A. flavicollis *mice and found an infection rate of 12.2% with *B. burgdorferi *s.l. Of 490 *I. ricinus *ticks feeding on rodents, 17 (3.5%) were infected with *B. burgdorferi *s.l., and *B. burgdorferi *s.l. was also detected in 15.3% (n = 262) of ticks feeding on blackbirds *Turdus merula *[Rosef, unpublished]. It seems that birds and rodents play an important role in maintaining *Borrelia *infection on Jomfruland. The prevalence of *B. burgdorferi *s.l. in ticks showed significantly lower values on Fjelløyvær than Jomfruland and Hinnebu than Jomfruland. In comparison there was no significance between Tjore and Jomfruland and Strøm and Jomfruland.

In contrast to infection with *B. burgdorferi *s.l., cervids are important reservoirs for *A. phagocytophilum*. Stuen et al. [13] found an overall high seroprevalence for *A. phagocytophilum *(formerly granulocytic *Ehrlichia *spp.) in moose, red deer and roe deer in Norway with 43%, 55% and 96% respectively. Experimental *Anaplasma *infection in red deer has shown subclinical persistent infection [37]. These wild ruminants are exposed to *A. phagocytophilum *and comprise the most widespread tick-borne infection in animals in Europe [38]. In Wisconsin, Michalski et al. [39] found a prevalence in ticks between 5.8% and 8.9%, and in white-tailed deer between 11.5% and 26% using PCR and DNA sequencing. A paretic condition in an *A. phagocytophilum *infected roe deer calf [40] and ehrlichiosis in a moose calf [12] has been observed in Norway. The high level of infected ticks at Fjelløyvær and Strøm (Table 4) not surprisingly shows that roe deer and red deer probably are competent reservoirs and vehicles for this bacterium.

A low prevalence of *A. phagocytophilum *in ticks from Hinnebu and Tjore was found in 2008 (Table 4) but it could not be detected in 2006 and 2007. The prevalence of *A. phagocytophilum *in host seeking *I. ricinus *ticks in Norway varied from zero to 19.4% in 18 sites investigated, with the highest prevalence occurring in Hitra [41]. The prevalence of *A. phagocytophilum *infections in ticks was significantly higher in localities with high density of wild cervids (Fjelløyvær and Strøm) compared to localities with lower densities (Tjore and Hinnebu) (Tables 1 and 4). An exception that cannot be explained occurred in 2008 when the prevalence of *A. phagocytophilum *was high in Tjore and low in Fjellværøy.

In Europe *B. burgdorferi *s.l. and *A. phagocytophilum *are transmitted by the same vector (*I. ricinus*), but it is unclear whether both pathogens use the same range of host species as reservoirs on a smaller scale. In Europe, studies conducted in the United Kingdom, Switzerland, Germany and the Czech Republic demonstrated that small rodents including *Myodes glareolus, Microtus arvalis, Microtus agrestis, Apodermus flavicollis *and *Apodermus sylvaticus *harbored *A. phagocytophilum *and were suggested as potential reservoirs [24,42-45]. In a study in Northern England, Bown et al. [42] described the maitainance of the enzootic cycle of *A. phagocytophilum *in the rodent -*I. trianguliceps *system. In a study conducted in Germany [43]* A. phagocytophilum *was detected in 13.4% of red bank voles and 6.2% of field voles. In contrast, only 0.5% of *A. flavicollis *was *A. phagocytophilum *positive. Investigations from Switzerland, England and Norway have shown that deer and sheep can be reservoir hosts [14,40,46]. Migrating birds have also been considered important in the dispersal of *A. phagocytophilum *infected *I. ricinus *in Europe and in the distribution of HGA [17,38].

*A. phagocytophilum *could not be detected in 49 rodents and in 24 *I. ricinus *nymphs feeding on rodents investigated on Jomfruland, possibly because *I. trianguliceps *is the main vector for *Anaplasma *in rodents [24,42]. *A. phagocytophilum *was found in ticks feeding on birds on Jomfruland [47]. This indicates that birds are involved in the maintenance of *Anaplasma *here, but rodents play only a minor role in the epidemiology of *Anaplasma *in the investigated areas in Norway. Hinnebu is located inland and is not on the main route of migrating birds. Tjore is near the coast, but not a typical site for migrating birds. Migrating birds, however, may play an important role as hosts for *I. ricinus *larvae and nymphs and probably for the infection route of *Anaplasma *(as for *B. burgdorferi *s.l.) [47]. On the island of Jomfruland the figures for *A. phagocytophilum *were 8.7%, 11.5% and 23% in 2006-2008. However, *A. phagocytophilum *was found on ticks feeding on birds in 33 out of 308 ticks investigated [47] on Jomfruland and also in questing ticks (Table 4). This indicates that birds are a possible reservoir. Both *B. burgdorferi *s.l. and *A. phagocytophilum *were found in ticks feeding on migrating birds and in questing ticks.

## Conclusion

A high prevalence of *A. phagocytophilum *in questing ticks in sites with high abundance of deer (>3 killed animals per km^2^) and low prevalence of *B. burgdorferi *s.l. was found, and we conclude that deer may be important reservoirs of *A. phagocytophilum *and incompetent carriers for *B. burgdorferi *s.l., thereby reducing the infection rate on questing *Ixodes ricinus *ticks.

## Competing interests

The authors declare that they have no competing interests.

## Authors' contributions

OR and AP have designed and performed the experimental study. OR has drafted the manuscript. JR has carried out the statistical and molecular genetic analyses. All authors read and approved the final manuscript
